# Paraguay’s Path Toward the Simplification of Procedures in the Approval of GE Crops

**DOI:** 10.3389/fbioe.2020.01023

**Published:** 2020-08-18

**Authors:** Nidia Benítez Candia, Danilo Fernández Ríos, Carmen Vicién

**Affiliations:** ^1^Departamento de Biotecnología, Facultad de Ciencias Exactas y Naturales, Universidad Nacional de Asunción, San Lorenzo, Paraguay; ^2^School of Agriculture, University of Buenos Aires, Buenos Aires, Argentina

**Keywords:** GE crops, regulatory system, acceptance of third-country assessments, simplified procedure, problem formulation

## Abstract

Agricultural biotechnology was first regulated in Paraguay in 1997. The first update to the country’s regulatory framework came in 2012, motivated by the need to keep up with current technologies. As part of this process, in late 2012, the Paraguayan Ministry of Agriculture (MAG) joined the Partnership for Biosafety Risk Assessment and Regulation, led by ILSI Research Foundation. The purpose of the program was the development of capacity building activities. As a result, the regulatory authorities in Paraguay incorporated the problem formulation approach to environmental risk assessment into their regulatory processes, leading to improved efficiency, with more timely decisions. Shifting to a problem formulation-based decision-making system was not straightforward, since practice and experience are always required to make professional risk assessors. Despite the continuity of approvals, there was a lag in the response time reflected in the number of events approved. During 2019, a simplified approval procedure for events that have been assessed by sound and experienced regulatory systems was introduced. Acceptance of third-country assessments can allow regulatory systems to make better use of their human, financial, and institutional resources, and stimulate inter-agency cooperation. In this work we aim to present the recent evolution of the regulatory system in Paraguay toward the establishment of a simplified procedure for GE crops that have been already assessed by sound and experienced regulatory systems, taking into account several scientific criteria. Concepts such as the familiarity, history of safe use, substantial equivalence, transportability, problem formulation, and the use of the consensus documents, developed by Organization for Economic Co-operation and Development (OECD), Food and Agriculture Organization of the United Nations (FAO), World Health Organization (WHO) and other institutions, favors the acceptance of decision documents issued by third countries. This requires the commitment of governments to support the stability of the institutions responsible for the regulatory implementation and also encourages countries to put work into the preparation and publication of decision documents, which are the basis for the commercialization of GE events.

## Introduction

Biosafety regulations around the world have evolved on a “piece by piece” basis, frequently in response to demands or needs of the moment (McLean et al., 2002). Consequently, the different levels of institutional development, and in particular of the innovative and educational systems, the different trade positions and the perception of societies about biotechnology, led to national strategies for the construction of regulatory systems, which, with some exceptions, were individual, without international coordination mechanisms (Vicién and Alvarez, 2013).

Furthermore, biosafety regulatory systems deal with evolving scientific knowledge and technologies, and thus inherently require constant adjustment of their procedures and requirements (Vicién and Trigo, 2017).

In that context, for several decades, international organizations like the Food and Agriculture Organization of the United Nations (FAO), the World Health Organization (WHO), and the Organization for Economic Co-operation and Development (OECD) have worked on the development of assessment criteria for food and feed derived from GE crops. The Codex Alimentarius Commission has established guidelines with the assessment criteria to be considered, which most countries follow. From the analysis of regulatory frameworks of different countries, considerable similarities were found in the type of information required: expression of new substances, analysis of allergenic or toxic potential, compositional analysis, impacts on the nutritional profile, among others; however, there are still differences regarding required data and methodologies. This heterogeneity, which is not always science-based, contributes to the complexity of the risk assessment process, thus making it longer and increasing costs (Fernández Ríos et al., 2019).

Concerning environmental risk assessment, the data collected in confined field trials consist of agro-phenotypic characteristics, which are used mainly to assess unintended effects (Ladics et al., 2015), and to confirm that there are no changes in reproductive biology or growth habits that could have an adverse environmental impact (Nakai et al., 2015). These data are compared with one or more comparators grown in the same trial as the transgenic plant, and the comparator is usually the untransformed or near-isogenic parental line (Clawson et al., 2019). In most cases, transgenic plants are evaluated in multi-location confined field trials in the country of origin over multiple growing seasons, and there may be no scientific rationale for conducting additional trials. If there is, then the risk hypotheses should be clearly articulated. Nevertheless, still, many countries routinely require in-country confined field trials, even when data from confined field trials in the country of origin are enough to prove environmental safety (Roberts, 2019). That being the case, it has to be remarked that even though not harmonized, regulatory requirements for environmental risk assessment are very similar between regulatory systems, as most of the concerns related to potential harms are consistently addressed across different countries (Center for Environmental Risk Assessment [CERA], 2012).

The agricultural sector is one of the economic pillars of Paraguay in its contribution to the GDP, with the main crops being soybean, cassava, maize, wheat, sugar cane, and cotton. It should also be noted that Paraguay is the world’s fourth exporter of soybean (MAG, 2020). The use of GE crops is important for the agricultural development of the country, making adequate access to products derived from biotechnology and its safe and sustainable incorporation to domestic production a vital requirement.

In 2020, the area planted with crops was 4.67 million hectares and consisted of soybean (3.56 million hectares), maize (1.08 million hectares), and cotton (18,000 hectares) (MAG, 2020). Since 2004, a total of 38 events^1^ were approved in Paraguay for food, feed, and cultivation use; including cotton, maize, and soybean events. According to ISAAA (2018), Paraguay is the sixth largest producer of GE crops. Almost 94% of the soybean, 36% of the maize, and 56% of the cotton planted in the country are GE.

Keeping that context in mind, in this work we aim to present the recent evolution of the regulatory system in Paraguay toward the establishment of a simplified procedure for GE crops that have been already assessed by transparent and experienced regulatory systems, taking into account several scientific criteria.

## The Beginnings

Agricultural biotechnology was first regulated in Paraguay in 1997. In 2012, the system was adjusted through the creation of the National Agricultural and Forestry Biosafety Commission (CONBIO), “with the mission to manage, analyze, and issue recommendations on all matters related to the introduction, confined field trials, pre-commercial and commercial release, and other intended uses of GE crops” (MAG, 2012).

One feature of GE crop applications for commercial release in Paraguay is that the transformation events have been in the global market for a while, and have thus been submitted to the scrutiny of regulatory systems that are sound and with experience in risk-assessment. There have been no applications for materials that are in the process of being developed locally or in a counter station development in the Northern Hemisphere.

Risk analysis followed a check-list criterion with exhaustive forms that did not clearly distinguish the differences between risk evaluation and risk management, despite having extensive information on approvals in third countries. There was a lack of a methodological framework on which to base the risk hypotheses that were applicable in the country’s conditions.

**FIGURE 1:**
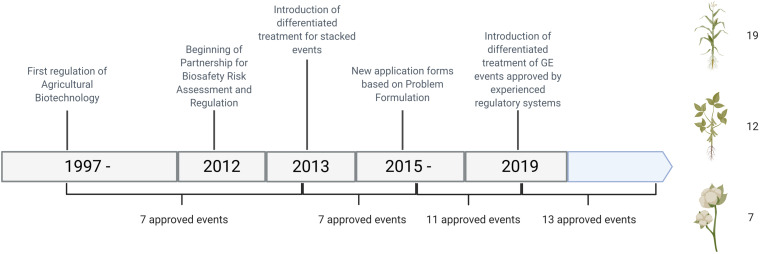
Number of GE event approvals in Paraguay from 2004 to 2019, divided by stage of development of the regulatory system.

The first transgenic crop was approved in 2004; 40-3-2/GTS40-3-2 Roundup Ready soybean. From 2004 to 2012, seven GE events were approved (Figure 1).

## Some Lessons From a Collaborative Program

In late 2012, the Paraguayan Ministry of Agriculture (MAG) joined the Partnership for Biosafety Risk Assessment and Regulation, by means of the signature of a Memorandum of Understanding between the National Agricultural and Forestry Commission and the International Life Science Institute (ILSI) Research Foundation. With the aim of strengthening the technical capacity of stakeholders in developing countries regarding biosafety risk assessment and regulation, this collaborative program was framed within a global project led by ILSI Research Foundation and funded by the World Bank (McLean and Roberts, 2015).

Through this partnership, ILSI developed a capacity building program for Paraguay, based on feedback received from Paraguayan government representatives and stakeholders in agricultural biotechnology. Suggestions and recommendations from participants were also incorporated along with the implementation phase of the program (Fernández Ríos et al., 2018).

Regarding the activities, they “included building a knowledge base focused on developing effective skills on Problem Formulation for ERA of GE crops with a hands-on approach; analysis on key elements and procedures of a regulatory system for confined field trials for each stage in the development cycle of a GE crop; special considerations to the cases of non-target organisms and stacked event crops and safety assessment of foods derived from GE plants” (Soerensen et al., 2014). Seminars and workshops on agricultural biotechnology aimed at a wider, interested audience, and specific working sessions for regulators, scientists and graduate students directly involved in risk assessment activities, with in-depth discussions of risk assessment concepts and tools, using a hands-on methodology were organized (McLean and Roberts, 2015; Fernández Ríos et al., 2018).

A critical factor for the program’s favorable outcome was the committed and coordinated effort of all participants from CONBIO, ILSI Research Foundation, and ILSI Argentina toward its implementation and the subsequent monitoring of its results. Other contributors were the National University of Asunción and the Argentine Council for Information and Development of Biotechnology (Argenbio), IICA’s office in Paraguay (Inter-American Institute for Cooperation on Agriculture), and the Institute of Agricultural Biotechnology in Paraguay (INBIO) (Soerensen et al., 2014).

The national regulatory authorities in Paraguay incorporated the problem formulation approach to environmental risk assessment into their regulatory processes, leading to an improvement in the regulatory system, which could be shown by the implementation of more timely decisions on the use of new GE crop varieties for commercial release. In this regard, “the time for decision making by the national regulatory authority was reduced from 2 years to 3 months” (McLean and Roberts, 2015). Between June 2013 and February 2014, seven GE events were approved.

In addition to this, a Ministerial Resolution dictated the differentiated treatment for stacked events whose parental lines had already been approved (MAG, 2013).

The unifying conceptual tools for the environmental and food/feed problem formulation-based risk assessment of GE crops (Wolt et al., 2010; Garcia-Alonso, 2013) were crucial to provide a firm scientific foundation to decision-making. Upon completion of the capacity building program, this deeper understanding of the scientific ground underlying biosafety regulation led to the development of science-based risk assessment guidelines and application forms for confined field trials and for commercial release of GE crops (which includes both food/feed and environmental evaluations), based on the problem formulation methodology (Soerensen et al., 2014; MAG, 2015).

## The Transition

The transition from the so-called “check-list” approach – applied from 1997 until 2012 in food/feed and environmental risk assessments – to one based on problem formulation was not a minor task, as the learning curve of the regulators and the time needed to adjust are generally underestimated. Despite having new guidelines for evaluating applications, the main issue was the integration of the problem formulation process within the risk assessment into everyday work and a clear identification of protection goals (Garcia-Alonso and Raybould, 2014).

In this regard, since the capacity building program ended in 2015, the program partners have implemented follow-up periodical meetings with the participants with a hands-on methodology to discuss particular topics or share new information, developments and publications in order to keep up improving the regulatory system (Fernández Ríos et al., 2018).

In spite of the program’s success in terms of capacity building and the follow-up implemented, CONBIO was still facing numerous difficulties. Its members are not fully dedicated, but due to the very nature of the composition of CONBIO they hold positions at institutions which they are designated to represent at CONBIO, and thus have other responsibilities derived from their positions at those institutions, lengthening assessment process. This fact showed the importance of having even a small group of dedicated risk assessors that could perform evaluations in a timely manner.

In addition, members were frequently replaced, and the advisors appointed by member institutions to be a part of CONBIO were experts in their respective fields, but quite rarely in risk assessment, which often generated debates about apprehensions that would not arise with a group specifically dedicated to and specialized in risk assessment (Fernández Ríos et al., 2018). It was difficult for these newly arrived members to adjust to analyzing information based on regulatory science criteria and examining dossiers as a source of data that responds to risk hypotheses. That leads to the consideration that practice and experience are always required to make professional risk assessors, and this is a lengthy process. These difficulties faced by CONBIO are rooted in its organizational structure, and thus would require organizational modifications or a simplification of operational procedures.

Between 2015 and 2018, eleven events were approved. Despite the continuity of approvals, the response time was lengthened, due largely to the issues indicated above.

## A Simplified Approval Procedure

In this context, in 2019 members of CONBIO considered and proposed the introduction of a simplified approval procedure for events that have been assessed by sound and experienced regulatory systems, thus maintaining the regular procedure for those GE crops that have not been previously assessed (MAG, 2015). The simplified procedure applies for commercial approvals hence including both food and feed and environmental evaluations. This implies the acceptance of scientific opinion by the regulatory authority in the country where the GE crop has been approved but only when several criteria have been taken into consideration in the risk assessment performed by those regulatory authorities.

Through MAG’s Resolutions 1030 and 1071 there was stated a differentiated treatment for the commercial release of novel GE crops and for GE crops that have been approved in third countries, whose scientific, technical and safety characteristics are well-founded (MAG, 2019a,b). As has already been indicated, Paraguay usually receives submissions to assess events that have been in the market for a while and have thus been submitted to the evaluation of regulatory systems that are sound and experienced. In addition, those countries usually share Paraguay’s protection goals.

Paraguayan Ministry of Agriculture’s Resolutions authorize taking into consideration the decision documents from third countries with regard to both human and animal food safety in the cases where these evaluations have been based on Codex Alimentarius, such as the Guidelines for the Conduct of Food Safety Assessment of Foods Derived from Recombinant-DNA Plants (Codex Alimentarius, 2003) and carried out in countries with time-tested regulatory systems and transparent procedures.

Concerning environmental safety, assessments are accepted for GE crops that besides having been authorized for commercial planting in countries with sound regulatory systems, include in the decision documents considerations as follows: that the GE crop under review has been studied under different environmental conditions, behaving in the same way as the conventional non-GE counterpart; that it will be managed in an agronomic manner similar to any GE or conventional hybrid/variety of the species; another aspect is that Paraguay is not center of origin of that crop, and finally two relevant characteristics are that there are no related weeds in Paraguay with which the GE crop could cross-breed and that the main target pests and the main non-target arthropod species present in Paraguay have been taken into account in the GE risk assessment carried out in those countries.

During 2019, in the period immediately following the adoption of the simplified procedure for events with commercial authorizations in third countries, thirteen events were approved; most of them with herbicide tolerance and/or Lepidoptera resistance, traits for which there is an extensive body of literature and experience with the safety of the novel proteins involved.

## Some Findings

So far, all applications for regulatory approvals in Paraguay have been for transformation events that were already in the global market, having been scrutinized by sound and experienced regulatory systems. There have been no requests to evaluate locally developed events. Besides, decision documents from said countries, where regulatory criteria are specified, have always been an important basis for the decision making in risk analysis in Paraguay. In other words, there is a history of using information and data from existing risk analyses, and the GE crops in consideration have been cultivated in a range of receiving environments. That is why it was considered appropriate to develop a simplified procedure that could allow regulatory authorities in Paraguay to focus human, financial and institutional resources in a manner that is commensurate with risk (Center for Environmental Risk Assessment [CERA], 2012). Figure 1 shows the number of approvals per period of development since the establishment of the regulatory system in Paraguay.

Prior to capacity-building activities, Paraguay approved events that had been cleared for marketing by an average of approximately eight countries and mostly consisted of single events. In the following period, from 2013 to 2014, events that had been launched commercially on average by seven countries were authorized. Again, most approvals were of single (non-stacked) events. From 2015 to 2018, which is the period just after the adoption of the new forms for commercial approval with a problem formulation approach, the country began to authorize mostly stacked events. Within this period, the events approved by Paraguay had previously been approved for commercialization on average by five countries. Finally, in the period immediately after the adoption of the simplified procedure for events with commercial planting authorizations in third countries, the events approved by Paraguay had previously been commercialized on average by three countries, again with a majority of stacked events approved.

Finally, GE crops approved in Paraguay through the simplified procedure were presented with prior approvals from Brazil (11 events), Argentina (8 events), Japan (7 events), Canada (5 events), United States (3 events). These regulatory systems are experienced, perform science-based food/feed and environmental risk assessments aided by the problem formulation approach, use consensus documents produced by the OECD and the Codex Alimentarius Commission, and have transparent GE event approval procedures.

## Final Remarks

Acceptance of third-country assessments can allow regulatory systems to make better use of their human, financial, and institutional resources, and stimulate inter-agency cooperation. As a first step toward acceptance, countries must have a clear understanding of the scientific grounds for the establishment of acceptance criteria. In the case of food safety, these criteria are sufficiently harmonized, which would facilitate acceptance. As for environmental risk assessments, the framework given by problem formulation methodology when reviewing decision documents is a basis for a common ground. It is always of the most crucial importance to develop proper risk hypotheses and to rely on regulators with solid backgrounds on risk-assessment. In addition, it is important to note that these processes will depend on the level of trust between the actors of the regulatory process, on the implementation of validated methodologies, and on the assurance of the quality and integrity of regulatory data.

Finally, several aspects must be considered by authorities of regulatory systems in order to incorporate procedures for the acceptance of decision documents from third countries. So that said procedures are to be appropriate to the regulatory system’s context, which means, will cause the least amount of disruption to the existing regulatory framework, will take into account the country’s protection goals and will be accepted/trusted by the public. Concepts such as the familiarity, history of safe use, substantial equivalence, transportability, problem formulation, and the use of the consensus documents, developed amongst others by OECD, FAO, WHO and other institutions, in turn, favors the establishment of the acceptance system. Nevertheless, this requires the commitment of governments to support the stability of the institutions responsible for the regulatory implementation and is also relevant that governments make an effort to prepare and publish decision documents which are the basis for authorizing commercialization of the events.

## Data Availability Statement

Publicly available datasets were analyzed in this study. The data can be found here: https://conbio.mag.gov.py/.

## Author Contributions

All authors participated in the drafting of this manuscript as individual experts in their fields, and are solely responsible for the contents.

## Disclaimer

Any views expressed in this manuscript are the views of the authors and do not necessarily represent the views of any organization, institution, or government to which they are affiliated or employed.

## Conflict of Interest

The authors declare that the research was conducted in the absence of any commercial or financial relationships that could be construed as a potential conflict of interest.

## Footnotes

1 Reports on the amount of approved GE events may vary depending on whether the parental lines and intermediate combinations approved through a single legal instrument are counted. For this work, we used the Biotrack Product Database (OECD) entry on Paraguay along with Decision documents from the Paraguayan government.
